# Limitations of Current GABA Agonists in Neonatal Seizures: Toward GABA Modulation Via the Targeting of Neuronal Cl^−^ Transport

**DOI:** 10.3389/fneur.2013.00078

**Published:** 2013-06-25

**Authors:** Arjun Khanna, Brian Patrick Walcott, Kristopher T. Kahle

**Affiliations:** ^1^Division of Neurosurgery, Department of Surgery, Massachusetts General Hospital, Harvard Medical School, Boston, MA, USA; ^2^Manton Center for Orphan Disease Research, Boston Children’s Hospital, Boston, MA, USA

**Keywords:** seizure, GABA, neonatal seizure, bumetanide, NKCC1, KCC2, WNK kinase, SPAK/OSR1 kinase

## Abstract

Neonatal intensive care has advanced rapidly in the last 40 years, with dramatic decreases in mortality and morbidity; however, for neonatal seizures, neither therapies nor outcomes have changed significantly. Basic and clinical studies indicate that seizures in neonates have long-term neurodevelopmental and psychiatric consequences, highlighting the need for novel pharmacotherapeutics. First-line treatments targeting GABAA receptors, like barbiturates and benzodiazepines, are limited in their efficacy and carry significant risks to the developing brain. Here, we review the use of current GABA agonist therapies for neonatal seizures and suggest other treatment strategies given recent developments in the understanding of disease pathogenesis. One promising avenue is the indirect manipulation of the GABAergic system, via the modulation of neuronal Cl^−^ gradients, by targeting the cation-Cl^−^ cotransporters (NKCC1 and KCC2) or their regulatory signaling molecules. This strategy might yield a novel class of more efficacious anti-epileptics with fewer side effects by specifically addressing disease pathophysiology. Moreover, this strategy may have ramifications for other adult seizure syndromes in which GABA receptor-mediated depolarizations play a pathogenic role, such as temporal lobe epilepsy.

## Current GABA Agonists and Prodrugs (Benzodiazepines and Barbiturates) are Inadequate in the Treatment of Neonatal Seizures

**Figure 1:**
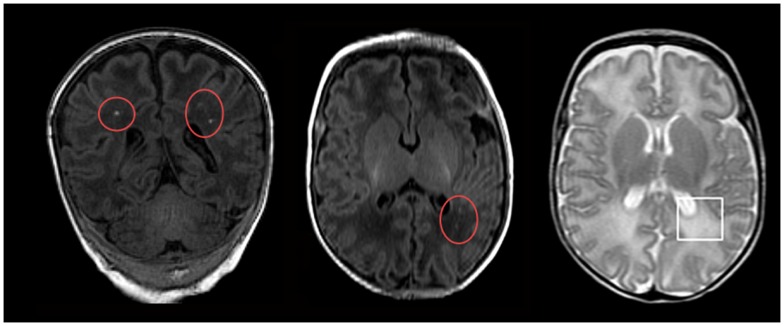
**Magnetic resonance imaging (MRI) of neonatal hypoxic-ischemia**. Neonatal hypoxic-ischemia is a common cause of white matter injury, the underlying factor in the development of neonatal seizures. These manifest on MRI as either punctate white matter lesions (red circles; seen on T1-weighted MRI imaging) or diffuse excessive high signal intensity (white square; seen on T2-weighted MRI imaging). Reprinted from Wisnowski et al. (2013) under the terms of the Creative Commons Attribution License.

Seizures are the most common neurological emergency in the neonate, with an estimated prevalence of 1.8–5 seizures/1000 live births in the US (Jensen, 2009). In fact, the lifespan risk of seizures is highest in the neonatal period. There are many conditions that may cause neonatal seizures, the most common of which is hypoxia/ischemia (Ronen et al., 1999; Tekgul et al., 2006) causing white matter injury (Figure 1). Seizures in the newborn can cause permanent and severe cognitive and behavioral abnormalities, as well as enhanced epileptogenicity later in life (Baram, 2003; Holmes, 2004; Swann, 2005), underlining the importance of effective therapy. Although neonatal seizures have a different etiology than adult seizures, current standard therapy involves compounds whose efficacy has only been well established in older patients. Benzodiazepines and barbiturates are the most common first-line therapies for neonatal seizures; however, the efficacy of these drugs in treating seizures in the newborn is not supported by robust evidence. The use of these drugs may have significant short- and long-term effects on brain development.

Current therapy for neonatal seizures is usually focused on early treatment with benzodiazepines and barbiturates. These drugs generally exert their effects via modulation of GABA action at the GABA_A_ receptor, or by antagonism of NMDA receptors. Although these drugs are effective in adults, they do not control neonatal seizures well. When given as monotherapy, phenobarbital controls seizures in less than half of newborns (Painter et al., 1999). In a study examining six neonates treated with second-line benzodiazepines, none responded to this medication (Boylan et al., 2004). A Cochrane review concluded that there was little evidence from the literature that supported the use of these drugs in treating neonatal seizures (Booth and Evans, 2004). Furthermore, some have suggested that benzodiazepines and barbiturates may resolve the outward clinical symptoms of seizures due to sedation without correcting underlying abnormal brain activity, exacerbating “electroclinical dissociation” between clinical symptoms and electrographic recording and leading to an overestimation of the true efficacy of these drugs (Boylan et al., 2002).

Developmental changes in the expression and activity of neurotransmitters and their receptors provide some insight into the basis of the increased susceptibility of the neonatal brain to seizures and the relative inefficacy of benzodiazepines and barbiturates in controlling them. Developmentally regulated ion channel configurations that facilitate easier depolarization and an increased reliance on glutamatergic transmission together increase the excitability of the neonatal brain, putatively to promote synaptogenesis, but simultaneously conferring a disposition to seizures (Rakhade and Jensen, 2009). Differences in GABA signaling in the immature brain also contribute to its overall enhanced excitability. GABA is the major inhibitory neurotransmitter in the adult central nervous system, but its receptors and synthetic enzyme do not reach maximal levels of expression until the fourth postnatal week in rats (Swann et al., 1989; Brooks-Kayal et al., 2001). Furthermore, while GABA hyperpolarizes adult neurons, it can depolarize neonatal neurons *in vivo* (Loturco et al., 1995). This may explain why benzodiazepines and barbiturates, which are GABA_A_ receptor agonists, have limited efficacy in controlling neonatal seizures.

## Short-Term Side Effects of Benzodiazepines and Barbiturates

In addition to limited efficacy, a growing body of evidence suggests that benzodiazepines and barbiturates cause short- and long-term harm to the developing neonatal brain. Cellular and neuromorphological effects of antiepileptic drugs have thus far only been extensively studied in rodent models, so further study is required to determine the acute neurotoxic effects of these drugs in humans.

In the neonatal rat, administration of any of the common antiepileptic drugs – phenytoin, phenobarbital, diazepam, clonazepam, vigabatrin, or valproate – all caused apoptotic neurodegeneration that was associated with reduced expression of neurotrophins (Bittigau et al., 2002). Importantly, these detrimental effects are consequences of the mechanism of action of these drugs and not cross-reactivity, pharmacokinetics, or other compound-specific toxicities. For example, apoptotic neurodegeneration is seen in phenobarbital and diazepam, but also in other anesthetics that agonize the GABA_A_ receptor (Jevtovic-Todorovic et al., 2003). Observations of apoptosis following anesthetics that agonize GABA_A_Rs have also been reported in non-human primates, suggesting a similar effect in humans. For example, isofluorane, whose mechanism of action includes activation of GABA_A_Rs, induces neuro-apoptosis in neonatal rhesus macaques (Brambrink et al., 2010, 2012).

The two most common classes of anti-convulsant drugs are GABA_A_ receptor agonists and NMDA receptor antagonists. Both NMDA receptor antagonists (Haberny et al., 2002; Hansen et al., 2004) and GABA_A_ receptor agonists (Bittigau et al., 2002) cause the activation of apoptotic pathways and down-regulate neurotrophins, which together cause apoptotic neurodegeneration and inhibit neurogenesis, particularly in regions of substantial neural growth in the early postnatal period such as the sub-ventricular zone of lateral ventricles, cerebellum, and the subgranular zone of the hippocampal dentate gyrus. Anticonvulsants that antagonize NMDA receptors or agonize GABA_A_ receptors cause significant changes in the neonatal rat cerebral cortex proteome as soon as 1 day following therapy, including deregulation of proteins associated with apoptosis, oxidative stress, inflammation, proliferation, and potentiation, suggesting a multitude of mechanisms by which such early exposure may result in neurodegeneration (Kaindl et al., 2008). Barbiturates may also alter the development of synaptic junctions (Dydyk and Rutczynski, 1980).

The adverse effects of antiepileptic drugs are age-dependent. The rodent brain undergoes a period of increased synaptogenesis, glial cell multiplication, myelination, and brain network reorganization in the first three postnatal weeks (Dobbing, 1974), during which the neonatal rat is most susceptible to developmental toxicity from antiepileptic drugs (Kaindl et al., 2008). The corresponding brain growth in humans begins during pregnancy and lasts up to the third postnatal year (Dobbing, 1974; Herschkowitz, 1988), also suggesting enhanced susceptibility to toxicity during this period.

**Figure 2:**
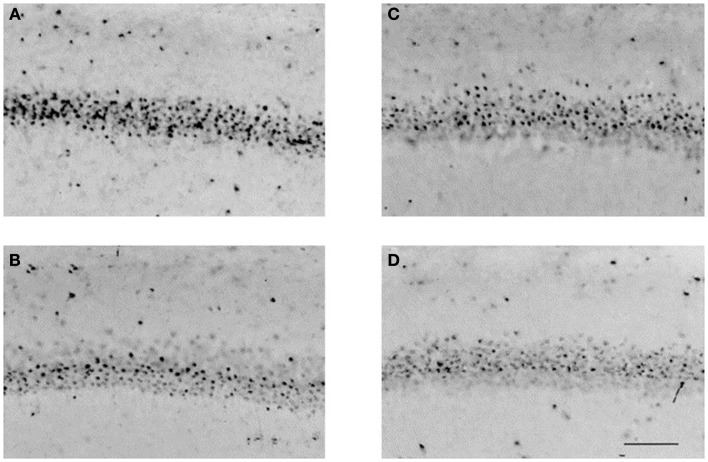
**GABA agonists affect the development of the neonatal brain**. Muscimol, a potent selective agonist for the GABA_A_ receptor, was administered to neonatal rats on postnatal days 0 and 1, and histologic specimens were obtained on postnatal day 21. Representative photomicrographs illustrate neuronal nuclear antigen-immunoreactive neurons in the CA1 region of the hippocampus in **(A)** sham males, **(B)** males + muscimol, **(C)** sham females, and **(D)** females + muscimol. Musciol treated rats have fewer neurons in the CA1 region of the hippocampus than their sham counterparts. Scale bar = 100 μm. Reprinted from Nunez et al. (2003), with permission from Elsevier.

The changes in cell proliferation and survival signaling pathways induced by common antiepileptic drugs likely contribute to altered brain morphology that is observed soon after developmental exposure (Figure 2). Decreased gray matter volume and altered gray matter morphology in a number of brain regions following developmental exposure to antiepileptic drugs is seen in rodents (Ikonomidou et al., 2007). Early barbiturate administration is consistently associated with decreased brain weight (Schain and Watanabe, 1975; Diaz et al., 1977) and neuronal number (Yanai et al., 1979) throughout rodent life, which is likely related to both apoptotic neurodegeneration and inhibited neurogenesis.

## Long-Term Side Effects of Benzodiazepines and Barbiturates

Determination of long-term cognitive and behavioral effects of early treatment with common anticonvulsants is difficult because cognition, behavior, and neuromorphology later in life is frequently heavily confounded by the condition for which anti-convulsant therapy was initially prescribed. However, there is general consensus that neonatal therapy with agents that interfere with GABA or glutamate transmission, including benzodiazepines, barbiturates, and phenytoin, cause cognitive and behavioral defects later in life. These cognitive and behavioral defects have been reported in both animal models and in humans.

Cognitive and behavioral deficits are likely caused by apoptotic neurodegeneration and inhibited neurogenesis in proliferative cells in the immature brain, and by changes in neural organization caused by the death of these neurons. Short-term drug-induced inhibition of neurogenesis can indirectly lead to destruction of pre-formed nerve populations and can thereby have a much broader impact on morphology later in development. For example, neonatal administration of phenobarbital led to a destruction of cerebellar and hippocampal granule cells, which form early in the postnatal period, as well as cerebellar Purkinje and hippocampal pyramidal neurons, which are formed prenatally, before phenobarbital was administered (Yanai et al., 1981). Since prenatally formed nerve cell populations are culled during synaptogenesis in normal brain development, barbiturate-induced destruction of presynaptic cells that ordinarily mature in the postnatal period can lead to a reduction in the number of prenatally formed postsynaptic neurons even without direct toxicity to these cells, since these pre-formed populations rely on synaptogenesis for survival through culling during development (Fishman and Yanai, 1983). In this way, even minor neurodegeneration or modulation of synaptogenesis during critical neurodevelopmental periods in the neonate can have major long-term effects on network organization and cognitive and behavioral deficits.

There is a wide array of reported cognitive and behavioral changes that are associated with early anticonvulsive therapy. Rats receiving phenobarbital show increased agitation, activity level, and aggression (Diaz and Schain, 1978). Adult rats that received early barbiturate therapy perform worse in cognitive tasks aimed at assessing hippocampal function (Yanai et al., 1989). Exposure to phenobarbital, phenytoin, or lamotrigine during the second postnatal week resulted in adult deficits in spatial learning, sensorimotor gating, and other behaviors in rats (Forcelli et al., 2012). The benzodiazepine diazepam has been shown to interfere with learning and memory in rats (Pereira et al., 1989; Holley et al., 1995). One study found that although phenobarbital therapy did reduce the number of seizures in developing rats, the cognitive deficits that resulted from phenobarbital therapy were greater in severity than the deficits in rats that did not receive phenobarbital, despite the fact that the latter group suffered more seizures (Mikati et al., 1994). Phenytoin therapy altered schedule-controlled behavior in rats that outlasted the therapy itself (Krafft et al., 1982).

Human studies also describe similar cognitive deficits. Children aged between 6 months to 3 years who were placed on 1 year of phenobarbital therapy after suffering from febrile seizures showed a negative correlation between serum levels of phenobarbital and memory concentration subscore of the Binet IQ test, although no significant difference in overall IQ or parent-reported behavior compared to controls was reported at 8 or 12 months of follow-up (Camfield et al., 1979). In a much larger study of 217 children aged between 8 and 36 months with febrile seizures, phenobarbital use significantly lowered IQ after 2 years compared to controls, but was not associated with a significant reduction in seizure recurrence (Farwell et al., 1990). Upon testing 3–5 years after medication had been stopped, early phenobarbital use significantly lowered performance in language/verbal tests and was associated with a reduction in IQ (Sulzbacher et al., 1999).

## Novel Targets of the GABAergic System: Targeting Molecules that Establish Cl^−^ Gradients

In the adult nervous system, due to low intracellular levels of Cl^−^, GABA inhibits most neurons by activation of GABA_A_Rs, causing Cl^−^ influx, membrane hyperpolarization, and inhibition. Additionally, GABA_A_R activation can also inhibit neurons via “shunting inhibition” by decreasing membrane resistance, which decreases the efficacy of excitatory signals to reach the critical threshold of action potential generation (Farrant and Kaila, 2007). Under several normal or pathophysiological circumstances, however, GABA can depolarize and even excite neurons rather than inhibit them. For example, in immature neurons during development and in certain neural subpopulations in pathophysiological states, GABA triggers depolarizations, and potential excitation (Cherubini et al., 1991; Ben-Ari et al., 1994), for review see (Marty and Llano, 2005). Although some have suggested that the observation of excitatory GABA responses is experimental artifact resulting from cellular damage in *ex vivo* studies (Bregestovski and Bernard, 2012), a large and growing body of evidence has confirmed excitatory GABA signaling in the developing brain *in vivo* and its physiological significance during development (Ben-Ari et al., 2012).

**Figure 3:**
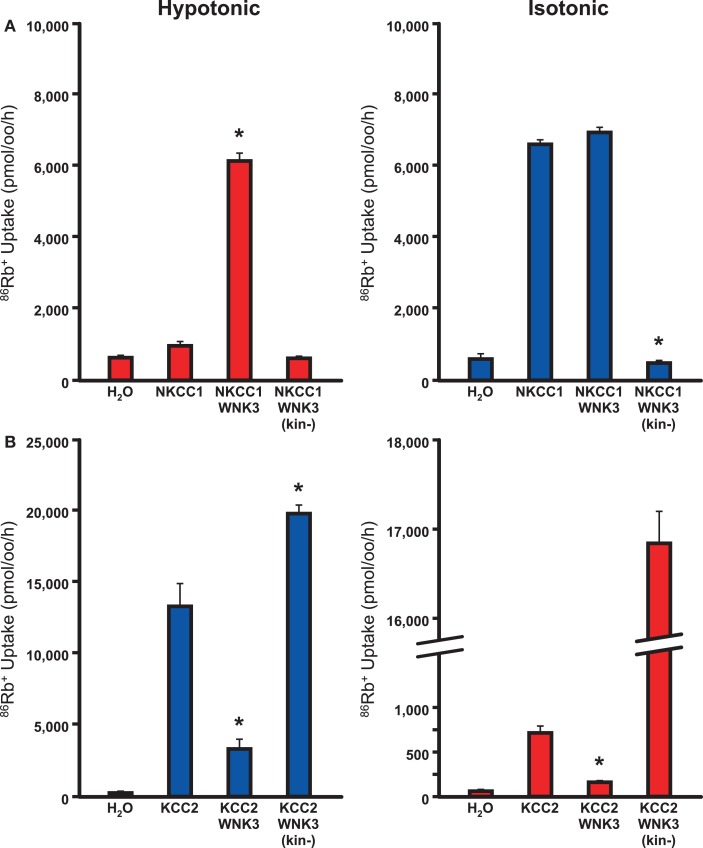
**WNK3 has opposite effects on NKCC1 and KCC2**. Xenopus oocytes were injected with cRNAs encoding NKCC1 **(A)** or KCC2 **(B)** alone and in combination with wild-type or kinase-dead mutant WNK3. 86Rb+ influx through NKCC1 **(A)** or efflux through KCC2 **(B)** was determined as a measure of flux through these cation Cl-channels. **(A)** WNK3 activates NKCC1. NKCC1 normally shows little activity under hypotonic conditions (180 mosM) and is active in isotonic conditions (200 mM). WNK3 increases NKCC1 to maximal activity in both conditions. Kinase-dead WNK3 strongly inhibits NKCC1 activity, indicating phosphorylation of NKCC1 by WNK3 is necessary for activity. *, *P* < 0.0001 vs. NKCC1 alone. **(B)** WNK3 inactivates KCC2. KCC2 is partially active under isotonic conditions and active under hypotonic conditions. WNK3 inhibits KCC2 under both conditions. Kinase-dead WNK3 strongly activates KCC2 under both hypotonic and isotonic conditions. *, *P* < 0.0001 vs. KCC2 alone. Taken together, these data suggest that WNK3 is an important regulator of NKCC1 and KCC2 activity. Its kinase activity activates NKCC1 and inhibits KCC2. Figure represents data from Kahle et al. (2005).

The depolarizing effect of GABA results from a change in the GABA_A_ reversal potential (E_GABA_) often secondary to an increased neuronal accumulation of Cl^−^, though ${\text{HCO}}_{3}^{-}$ also plays a role. Several notable ion channels and transporters are integral for neuronal Cl^−^ homeostasis. Among these are the bumetanide-sensitive Na^+^-K^+^-Cl^−^co-transporter NKCC1, which transports Cl^−^into the cell, and KCC2, which normally extrudes it (Kahle et al., 2008b; Blaesse et al., 2009) (Figure 3).

In immature neurons, the Cl^−^-exporting activity of KCC2 is lower than in mature neurons (Rivera et al., 1999), and in the context of NKCC1 expression, neuronal [Cl^−^]_i_ is higher and E_GABA_ is more depolarized (Figure 3B). Moreover, long runs of action potentials in single neurons (Woodin et al., 2003), or prolonged seizures may result in an activity-dependent accumulation of intracellular Cl^−^ (Khalilov et al., 2003; Dzhala et al., 2010). These data might provide insight as to why traditional anti-seizure medications are less effective when given later in the course of neonatal seizures (Painter et al., 1999).

Ion transport proteins that establish Cl^−^ gradients across neuronal membranes and contribute to depolarizing GABA responses in the immature brain could be novel targets for modulation of the GABAergic system in treating neonatal seizures (Kahle et al., 2008b). The commonly used loop diuretic bumetanide has a higher affinity for NKCC1 than KCC2 at low concentrations (Isenring et al., 1998; Hannaert et al., 2002). In a human neonate in *status epilepticus* (Kahle et al., 2009) and in animal models of *status epilepticus* (Dzhala et al., 2005), bumetanide reduces electrographic seizures and exhibits synergy with phenobarbital, which increases the open probability of the GABA_A_ channel (Dzhala et al., 2008).

While several studies have reported anti-convulsant effects of bumetanide (Schwartzkroin et al., 1998; Reid et al., 2000; Mazarati et al., 2009), others have found no significant anti-convulsant effect (Ostergaard et al., 1972; Mares, 2009; Minlebaev and Khazipov, 2011). Most of the studies that demonstrate efficacy of bumetanide were performed when it was administered with an anesthetic or other drug that has GABA-modulatory effects, like phenobarbital. Other NKCC1 inhibitors with greater blood-brain-barrier penetration may have improved efficacy. Bumetanide pro-drugs that mask the hydrophilic acid group with esters to facilitate transport into the brain before releasing the active molecule are currently being tested (Loscher et al., 2013).

Currently there are two clinical trials evaluating bumetanide as a treatment for neonatal seizures. A phase I trial (NCT00830531) is actively enrolling patients in a randomized, double-blind, controlled dose-escalation study of bumetanide as an add-on therapy to treat refractory seizures caused by hypoxic-ischemic encephalopathy. The estimated study completion date is December 2015. A second trial (NCT01434225) is being performed by a large, multi-center European group in an “open-label,” dose-escalation fashion to assess the effect of bumetanide in addition to phenobarbital for the treatment of neonatal seizures caused by hypoxic-ischemic encephalopathy. Data from these pilot studies will be utilized to guide the design of larger Phase III trials that will determine the efficacy of bumetanide in the treatment of neonatal seizures.

**Figure 4:**
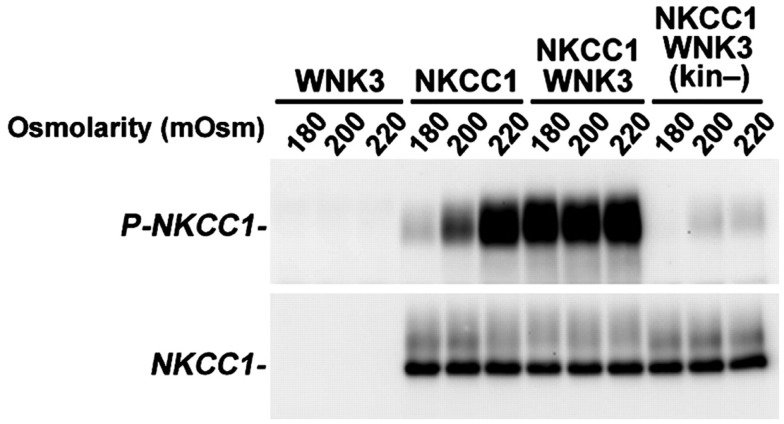
**Inhibition of WNK3 kinase activity potently reduces stimulatory phosphorylation of NKCC1**. WNK3 regulates the phosphorylation of NKCC1. *Xenopus* oocytes were injected with the indicated cRNAs and incubated at varying extracellular osmolarities (180, 200, and 220 mOsm). After incubation, oocytes were lysed, and Western blotting was performed with probes for phosphorylated NKCC1 (P-NKCC1) or unphosphorylated NKCC1 (NKCC1). Phosphorylation of NKCC1 normally increases from negligible levels in hypotonic conditions (180 mOsm) to complete phosphorylation in hypertonic conditions (220 mOsm). Coexpression of NKCC1 with kinase-active WNK3 results in robust phosphorylation of NKCC1 at all osmolarities, but coexpression with kinase-dead WNK3 results in marked reduction of NKCC1 phosphorylation at all osmolarities. Reprinted from Kahle et al. (2005), under their policy that allows for the use of this figure in this review. Copyright, 2005, The National Academy of Sciences.

There are some concerns about the modulation of depolarizing GABA responses based on its importance during neurodevelopment. Studies suggest the “premature” shift of E_GABA_ via NKCC1-knockdown or KCC2-overexpression may result in cortical neurons with fewer and shorter dendrites (Cancedda et al., 2007), less mature dendritic spines with decreased density and increased lengths, and fewer glutamatergic inputs received by cortical pyramidal neurons (Wang and Kriegstein, 2008). Some of these observations have also been recapitulated in rats (Wang and Kriegstein, 2011). More rigorous, long-term follow-up in neonates receiving bumetanide therapy is required, including comparison with current therapies, to determine neuropsychiatric sequelae associated with modulation of neuronal Cl^−^ gradients in GABA transmission. Nevertheless, in the context of our enhanced understanding of the mechanisms of GABA signaling in the neonatal brain, novel therapies that target Cl^−^ homeostasis are promising new targets for the treatment of neonatal seizures (Loscher et al., 2013). Alternative mechanisms to modulate neuronal Cl^−^ homeostasis should also be considered. One unexplored mechanism of Cl^−^ modulation is the manipulation of neuronal Cl^−^ gradients via targeting of the serine-threonine regulatory kinases of NKCC1 and KCC2 (Kahle et al., 2010). For example, phosphorylation of NKCC1 at critical N-terminal threonine residues is required for transporter activity, and this event is catalyzed by the WNK/SPAK kinase cascade (for review, see Kahle et al., 2008a). Inhibition of WNK3 kinase activity, a molecule expressed highly in the developing brain, is the most potent means of concurrently inhibiting NKCC1 activity and activating KCC2 activity *in vitro*, and does so via changes in transporter phosphorylation at critical regulatory residues (Figure 4) (Kahle et al., 2005; de Los Heros et al., 2006). Given the coordinated but reciprocal stimulatory and inhibitory effect of phosphorylation on NKCC1 and KCC2, respectively, we propose that inhibition of the WNK/SPAK pathway in neurons might help restore GABA inhibition. These hypotheses are currently being explored in neuronal culture systems and whole animal models of seizures in multiple labs, including our own. These efforts may prove useful not only for neonatal seizure management, but also for adult seizure syndromes that exhibit depolarized GABA responses and altered Cl^−^ homeostasis, such as temporal lobe epilepsy, in which abnormal expression of Cl^−^ channels also renders GABA excitatory (Palma et al., 2006; Huberfeld et al., 2007; Munoz et al., 2007; Brandt et al., 2010; Maa et al., 2011; Miles et al., 2012; Eftekhari et al., 2013).

## Conflict of Interest Statement

The authors declare that the research was conducted in the absence of any commercial or financial relationships that could be construed as a potential conflict of interest.

## References

[B1] Baram T. Z. (2003). Long-term neuroplasticity and functional consequences of single versus recurrent early-life seizures. Ann. Neurol. 54, 701–705 10.1002/ana.10833 14681879 PMC2981791

[B2] Ben-Ari Y. Tseeb V. Raggozzino D. Khazipov R. Gaiarsa J. L. (1994). γ-Aminobutyric acid (GABA): a fast excitatory transmitter which may regulate the development of hippocampal neurones in early postnatal life. Prog. Brain Res. 102, 261–273 10.1016/S0079-6123(08)60545-2 7800817

[B3] Ben-Ari Y. Woodin M. A. Sernagor E. Cancedda L. Vinay L. Rivera C. (2012). Refuting the challenges of the developmental shift of polarity of GABA actions: GABA more exciting than ever! Front. Cell. Neurosci. 6:35 10.3389/fncel.2012.00035 22973192 PMC3428604

[B4] Bittigau P. Sifringer M. Genz K. Reith E. Pospischil D. Govindarajalu S. (2002). Antiepileptic drugs and apoptotic neurodegeneration in the developing brain. Proc. Natl. Acad. Sci. U.S.A. 99, 15089–15094 10.1073/pnas.222550499 12417760 PMC137548

[B5] Blaesse P. Airaksinen M. S. Rivera C. Kaila K. (2009). Cation-chloride cotransporters and neuronal function. Neuron 61, 820–838 10.1016/j.neuron.2009.03.003 19323993

[B6] Booth D. Evans D. J. (2004). Anticonvulsants for neonates with seizures. Cochrane Database Syst. Rev. 3:CD004218 10.1002/14651858.CD004218.pub2 15495087 PMC12536760

[B7] Boylan G. B. Rennie J. M. Chorley G. Pressler R. M. Fox G. F. Farrer K. (2004). Second-line anticonvulsant treatment of neonatal seizures: a video-EEG monitoring study. Neurology 62, 486–488 10.1212/01.WNL.0000106944.59990.E6 14872039

[B8] Boylan G. B. Rennie J. M. Pressler R. M. Wilson G. Morton M. Binnie C. D. (2002). Phenobarbitone, neonatal seizures, and video-EEG. Arch. Dis. Child. Fetal Neonatal Ed. 86, F165–F170 10.1136/fn.86.3.F165 11978746 PMC1721395

[B9] Brambrink A. M. Back S. A. Riddle A. Gong X. Moravec M. D. Dissen G. A. (2012). Isoflurane-induced apoptosis of oligodendrocytes in the neonatal primate brain. Ann. Neurol. 72, 525–535 10.1002/ana.23652 23109147 PMC3490441

[B10] Brambrink A. M. Evers A. S. Avidan M. S. Farber N. B. Smith D. J. Zhang X. (2010). Isoflurane-induced neuroapoptosis in the neonatal rhesus macaque brain. Anesthesiology 112, 834–841 10.1097/ALN.1090b1013e3181d1049cd 20234312 PMC3962067

[B11] Brandt C. Nozadze M. Heuchert N. Rattka M. Loscher W. (2010). Disease-modifying effects of phenobarbital and the NKCC1 inhibitor bumetanide in the pilocarpine model of temporal lobe epilepsy. J. Neurosci. 30, 8602–8612 10.1523/JNEUROSCI.0633-10.2010 20573906 PMC6634618

[B12] Bregestovski P. Bernard C. (2012). Excitatory GABA: how a correct observation may turn out to be an experimental artifact. Front. Pharmacol. 3:65 10.3389/fphar.2012.00065 22529813 PMC3329772

[B13] Brooks-Kayal A. R. Shumate M. D. Jin H. Rikhter T. Y. Kelly M. E. Coulter D. A. (2001). γ-Aminobutyric acidA receptor subunit expression predicts functional changes in hippocampal dentate granule cells during postnatal development. J. Neurochem. 77, 1266–1278 10.1046/j.1471-4159.2001.00329.x 11389177

[B14] Camfield C. S. Chaplin S. Doyle A.-B. Shapiro S. H. Cummings C. Camfield P. R. (1979). Side effects of phenobarbital in toddlers; behavioral and cognitive aspects. J. Pediatr. 95, 361–365 10.1016/S0022-3476(79)80507-7 381616

[B15] Cancedda L. Fiumelli H. Chen K. Poo M.-M. (2007). Excitatory GABA action is essential for morphological maturation of cortical neurons in vivo. J. Neurosci. 27, 5224–5235 10.1523/JNEUROSCI.5169-06.2007 17494709 PMC6672363

[B16] Cherubini E. Gaiarsa J. L. Ben-Ari Y. (1991). GABA: an excitatory transmitter in early postnatal life. Trends Neurosci. 14, 515–519 10.1016/0166-2236(91)90003-D 1726341

[B17] de Los Heros P. Kahle K. T. Rinehart J. Bobadilla N. A. Vazquez N. San Cristobal P. (2006). WNK3 bypasses the tonicity requirement for K-Cl cotransporter activation via a phosphatase-dependent pathway. Proc. Natl. Acad. Sci. U.S.A. 103, 1976–1981 10.1073/pnas.0510947103 16446421 PMC1413675

[B18] Diaz J. Schain R. J. (1978). Phenobarbital: effects of long-term administration on behavior and brain of artificially reared rats. Science 199, 90–91 10.1126/science.199.4324.90 17569495

[B19] Diaz J. Schain R. J. Bailey B. G. (1977). Phenobarbital-induced brain growth retardation in artificially reared rat pups. Biol. Neonate 32, 77–82 10.1159/000240998 901883

[B20] Dobbing J. (1974). The later growth of the brain and its vulnerability. Pediatrics 53, 2–6 4588131

[B21] Dydyk L. Rutczynski M. (1980). Development of neuropil and synaptic junctions in brain cortex and stem of rabbit newborns after the transplacental action of luminal. Acta Med. Pol. 21, 1–10 7405623

[B22] Dzhala V. I. Brumback A. C. Staley K. J. (2008). Bumetanide enhances phenobarbital efficacy in a neonatal seizure model. Ann. Neurol. 63, 222–235 10.1002/ana.21229 17918265

[B23] Dzhala V. I. Kuchibhotla K. V. Glykys J. C. Kahle K. T. Swiercz W. B. Feng G. (2010). Progressive NKCC1-dependent neuronal chloride accumulation during neonatal seizures. J. Neurosci. 30, 11745–11761 10.1523/JNEUROSCI.1769-10.2010 20810895 PMC3070296

[B24] Dzhala V. I. Talos D. M. Sdrulla D. A. Brumback A. C. Mathews G. C. Benke T. A. (2005). NKCC1 transporter facilitates seizures in the developing brain. Nat. Med. 11, 1205–1213 10.1038/nm1301 16227993

[B25] Eftekhari S. Mehvari Habibabadi J. Najafi Ziarani M. Hashemi Fesharaki S. S. Gharakhani M. Mostafavi H. (2013). Bumetanide reduces seizure frequency in patients with temporal lobe epilepsy. Epilepsia 54, e9–12 10.1111/j.1528-1167.2012.03654.x 23061490

[B26] Farrant M. Kaila K. (2007). “The cellular, molecular and ionic basis of GABAA receptor signalling,” in Progress in Brain Research, eds James E. D. A. Tepper M. Bolam J. P. (London: Elsevier), 59–87 10.1016/S0079-6123(06)60005-8 17499109

[B27] Farwell J. R. Lee Y. J. Hirtz D. G. Sulzbacher S. I. Ellenberg J. H. Nelson K. B. (1990). Phenobarbital for febrile seizures – effects on intelligence and on seizure recurrence. N. Engl. J. Med. 322, 364–369 10.1056/NEJM199002083220604 2242106

[B28] Fishman R. H. B. Yanai J. (1983). Long-lasting effects of early barbiturates on central nervous system and behavior. Neurosci. Biobehav. Rev. 7, 19–28 6132355 10.1016/0149-7634(83)90004-0

[B29] Forcelli P. A. Kozlowski R. Snyder C. Kondratyev A. Gale K. (2012). Effects of neonatal antiepileptic drug exposure on cognitive, emotional, and motor function in adult rats. J. Pharmacol. Exp. Ther. 340, 558–566 10.1124/jpet.111.188862 22129597 PMC3286323

[B30] Haberny K. A. Paule M. G. Scallet A. C. Sistare F. D. Lester D. S. Hanig J. P. (2002). Ontogeny of the N-methyl-d-aspartate (NMDA) receptor system and susceptibility to neurotoxicity. Toxicol. Sci. 68, 9–17 10.1093/toxsci/68.1.9 12075105

[B31] Hannaert P. H. Alvarez-Guerra M.-G. Pirot D. P. Nazaret C. N. Garay R. G. (2002). Rat NKCC2/NKCC1 cotransporter selectivity for loop diuretic drugs. Naunyn Schmiedebergs Arch. Pharmacol. 365, 193–199 10.1007/s00210-001-0521-y 11882915

[B32] Hansen H. H. Briem T. Dzietko M. Sifringer M. Voss A. Rzeski W. (2004). Mechanisms leading to disseminated apoptosis following NMDA receptor blockade in the developing rat brain. Neurobiol. Dis. 16, 440–453 10.1016/j.nbd.2004.03.013 15193300

[B33] Herschkowitz N. (1988). Brain development in the fetus, neonate and infant. Neonatology 54, 1–19 10.1159/000242818 3061475

[B34] Holley L. Turchi J. Apple C. Sarter M. (1995). Dissociation between the attentional effects of infusions of a benzodiazepine receptor agonist and an inverse agonist into the basal forebrain. Psychopharmacology (Berl.) 120, 99–108 10.1007/BF02246150 7480541

[B35] Holmes G. L. (2004). Effects of early seizures on later behavior and epileptogenicity. Ment. Retard. Dev. Disabil. Res. Rev. 10, 101–105 10.1002/mrdd.20019 15362164

[B36] Huberfeld G. Wittner L. Clemenceau S. Baulac M. Kaila K. Miles R. (2007). Perturbed chloride homeostasis and GABAergic signaling in human temporal lobe epilepsy. J. Neurosci. 27, 9866–9873 10.1523/JNEUROSCI.2761-07.2007 17855601 PMC6672644

[B37] Ikonomidou C. Scheer I. Wilhelm T. Juengling F. D. Titze K. Stöver B. (2007). Brain morphology alterations in the basal ganglia and the hypothalamus following prenatal exposure to antiepileptic drugs. Eur. J. Paediatr. Neurol. 11, 297–301 10.1016/j.ejpn.2007.02.006 17418601

[B38] Isenring P. Jacoby S. C. Chang J. Forbush B. (1998). Mutagenic mapping of the Na-K-Cl cotransporter for domains involved in ion transport and bumetanide binding. J. Gen. Physiol. 112, 549–558 10.1085/jgp.112.5.549 9806964 PMC2229443

[B39] Jensen F. E. (2009). Neonatal seizures: an update on mechanisms and management. Clin. Perinatol. 36, 881–900 10.1016/j.clp.2009.08.001 19944840 PMC2818833

[B40] Jevtovic-Todorovic V. Hartman R. E. Izumi Y. Benshoff N. D. Dikranian K. Zorumski C. F. (2003). Early exposure to common anesthetic agents causes widespread neurodegeneration in the developing rat brain and persistent learning deficits. J. Neurosci. 23, 876–882 12574416 10.1523/JNEUROSCI.23-03-00876.2003 PMC6741934

[B41] Kahle K. T. Barnett S. M. Sassower K. C. Staley K. J. (2009). Decreased seizure activity in a human neonate treated with bumetanide, an inhibitor of the Na^+^-K^+^-2Cl^−^cotransporter NKCC1. J. Child Neurol. 24, 572–576 10.1177/0883073809333526 19406757

[B42] Kahle K. T. Rinehart J. De Los Heros P. Louvi A. Meade P. Vazquez N. (2005). WNK3 modulates transport of Cl^−^ in and out of cells: implications for control of cell volume and neuronal excitability. Proc. Natl. Acad. Sci. U.S.A. 102, 16783–16788 10.1073/pnas.0508307102 16275911 PMC1283843

[B43] Kahle K. T. Rinehart J. Lifton R. P. (2010). Phosphoregulation of the Na–K–2Cl and K–Cl cotransporters by the WNK kinases. Biochim. Biophys. Acta 1802, 1150–1158 10.1016/j.bbadis.2010.07.009 20637866 PMC3529164

[B44] Kahle K. T. Ring A. M. Lifton R. P. (2008a). Molecular physiology of the WNK kinases. Annu. Rev. Physiol. 70, 329–355 10.1146/annurev.physiol.70.113006.100651 17961084

[B45] Kahle K. T. Staley K. J. Nahed B. V. Gamba G. Hebert S. C. Lifton R. P. (2008b). Roles of the cation-chloride cotransporters in neurological disease. Nat. Clin. Pract. Neurol. 4, 490–503 10.1038/ncpneuro0883 18769373

[B46] Kaindl A. M. Koppelstaetter A. Nebrich G. Stuwe J. Sifringer M. Zabel C. (2008). Brief alteration of NMDA or GABAA receptor-mediated neurotransmission has long term effects on the developing cerebral cortex. Mol. Cell. Proteomics 7, 2293–2310 18587059 10.1074/mcp.M800030-MCP200

[B47] Khalilov I. Holmes G. L. Ben-Ari Y. (2003). In vitro formation of a secondary epileptogenic mirror focus by interhippocampal propagation of seizures. Nat. Neurosci. 6, 1079–1085 10.1038/nn1125 14502289

[B48] Krafft K. Lyon D. O. Poling A. (1982). Effects of phenytoin on schedule-controlled performance of rats. Psychopharmacology (Berl.) 78, 93–95 10.1007/BF00470597 6815703

[B49] Loscher W. Puskarjov M. Kaila K. (2013). Cation-chloride cotransporters NKCC1 and KCC2 as potential targets for novel antiepileptic and antiepileptogenic treatments. Neuropharmacology 69, 62–74 10.1016/j.neuropharm.2012.05.045 22705273

[B50] Loturco J. J. Owens D. F. Heath M. J. S. Davis M. B. E. Kriegstein A. R. (1995). GABA and glutamate depolarize cortical progenitor cells and inhibit DNA synthesis. Neuron 15, 1287–1298 10.1016/0896-6273(95)90008-X 8845153

[B51] Maa E. H. Kahle K. T. Walcott B. P. Spitz M. C. Staley K. J. (2011). Diuretics and epilepsy: will the past and present meet? Epilepsia 52, 1559–1569 10.1111/j.1528-1167.2011.03203.x 21838793

[B52] Mares P. (2009). Age- and dose-specific anticonvulsant action of bumetanide in immature rats. Physiol. Res. 58, 927–930 20059292 10.33549/physiolres.931897

[B53] Marty A. Llano I. (2005). Excitatory effects of GABA in established brain networks. Trends Neurosci. 28, 284–289 10.1016/j.tins.2005.04.003 15927683

[B54] Mazarati A. Shin D. Sankar R. (2009). Bumetanide inhibits rapid kindling in neonatal rats. Epilepsia 50, 2117–2122 10.1111/j.1528-1167.2009.02048.x 19260939 PMC2732750

[B55] Mikati M. A. Holmes G. L. Chronopoulos A. Hyde P. Thurber S. Gatt A. (1994). Phenobarbital modifies seizure-related brain injury in the developing brain. Ann. Neurol. 36, 425–433 10.1002/ana.410360314 8080250

[B56] Miles R. Blaesse P. Huberfeld G. Wittner L. Kaila K. (2012). “Chloride homeostasis and GABA signaling in temporal lobe epilepsy,” in Jasper’s Basic Mechanisms of the Epilepsies, 4th Edn, eds Noebels J. L. Avoli M. Rogawski M. A. Olsen R. W. Delgado-Escueta A. V. (Bethesda: National Center for Biotechnology Information), 581–591 22787654

[B57] Minlebaev M. Khazipov R. (2011). Antiepileptic effects of endogenous beta-hydroxybutyrate in suckling infant rats. Epilepsy Res. 95, 100–109 10.1016/j.eplepsyres.2011.03.003 21470827

[B58] Munoz A. Mendez P. Defelipe J. Alvarez-Leefmans F. J. (2007). Cation-chloride cotransporters and GABA-ergic innervation in the human epileptic hippocampus. Epilepsia 48, 663–673 10.1111/j.1528-1167.2007.00986.x 17319917

[B59] Nunez J. L. Alt J. J. McCarthy M. M. (2003). A novel model for prenatal brain damage. II. Long-term deficits in hippocampal cell number and hippocampal-dependent behavior following neonatal GABA_A_ receptor activation. Exp. Neurol. 181, 270–280 10.3201/eid0906.020377 PMC3000133 12781999

[B60] Ostergaard E. H. Magnussen M. P. Nielsen C. K. Eilertsen E. Frey H. H. (1972). Pharmacological properties of 3-n-butylamino-4-phenoxy-5-sulfamylbenzoic acid (Bumetanide), a new potent diuretic. Arzneimittel-Forschung 22, 66–72 5067001

[B61] Painter M. J. Scher M. S. Stein A. D. Armatti S. Wang Z. Gardiner J. C. (1999). Phenobarbital compared with phenytoin for the treatment of neonatal seizures. N. Engl. J. Med. 341, 485–489 10.1056/NEJM199908123410704 10441604

[B62] Palma E. Amici M. Sobrero F. Spinelli G. Di Angelantonio S. Ragozzino D. (2006). Anomalous levels of Cl^−^ transporters in the hippocampal subiculum from temporal lobe epilepsy patients make GABA excitatory. Proc. Natl. Acad. Sci. U.S.A. 103, 8465–8468 10.1073/pnas.0602979103 16709666 PMC1482515

[B63] Pereira M. E. Rosat R. Huang C. H. Godoy M. G. C. Izquierdo I. (1989). Inhibition by diazepam of the effect of additional training and of extinction on the retention of shuttle avoidance behavior in rats. Behav. Neurosci. 103, 202–205 10.1037/0735-7044.103.1.202 2923673

[B64] Rakhade S. N. Jensen F. E. (2009). Epileptogenesis in the immature brain: emerging mechanisms. Nat. Rev. Neurol. 5, 380–391 10.1038/nrneurol 19578345 PMC2822660

[B65] Reid K. H. Guo S. Z. Iyer V. G. (2000). Agents which block potassium-chloride cotransport prevent sound-triggered seizures in post-ischemic audiogenic seizure-prone rats. Brain Res. 864, 134–137 10.1016/S0006-8993(00)02121-1 10793196

[B66] Rivera C. Voipio J. Payne J. A. Ruusuvuori E. Lahtinen H. Lamsa K. (1999). The K^+^/Cl^−^ co-transporter KCC2 renders GABA hyperpolarizing during neuronal maturation. Nature 397, 251–255 10.1038/16697 9930699

[B67] Ronen G. M. Penney S. Andrews W. (1999). The epidemiology of clinical neonatal seizures in Newfoundland: a population-based study. J. Pediatr. 134, 71–75 10.1016/S0022-3476(99)70374-4 9880452

[B68] Schain R. J. Watanabe K. (1975). Effect of chronic phenobarbital administration upon brain growth of the infant rat. Exp. Neurol. 47, 509–515 10.1016/0014-4886(75)90083-7 1132462

[B69] Schwartzkroin P. A. Baraban S. C. Hochman D. W. (1998). Osmolarity, ionic flux, and changes in brain excitability. Epilepsy Res. 32, 275–285 10.1016/S0920-1211(98)00058-8 9761327

[B70] Sulzbacher S. Farwell J. R. Temkin N. Lu A. S. Hirtz D. G. (1999). Late cognitive effects of early treatment with phenobarbital. Clin. Pediatr. (Phila.) 38, 387–394 10.1177/000992289903800702 10416094

[B71] Swann J. W. (2005). The impact of seizures on developing hippocampal networks. Prog. Brain Res. 147, 347–354 10.1016/S0079-6123(04)47024-1 15581716

[B72] Swann J. W. Brady R. J. Martin D. L. (1989). Postnatal development of GABA-mediated synaptic inhibition in rat hippocampus. Neuroscience 28, 551–561 10.1016/0306-4522(89)90004-3 2710330

[B73] Tekgul H. Gauvreau K. Soul J. Murphy L. Robertson R. Stewart J. (2006). The current etiologic profile and neurodevelopmental outcome of seizures in term newborn infants. Pediatrics 117, 1270–1280 10.1542/peds.2005-1178 16585324

[B74] Wang D. D. Kriegstein A. R. (2008). GABA regulates excitatory synapse formation in the neocortex via NMDA receptor activation. J. Neurosci. 28, 5547–5558 10.1523/JNEUROSCI.5599-07.2008 18495889 PMC2684685

[B75] Wang D. D. Kriegstein A. R. (2011). Blocking early GABA depolarization with bumetanide results in permanent alterations in cortical circuits and sensorimotor gating deficits. Cereb. Cortex 21, 574–587 10.1093/cercor/bhq124 20624842 PMC3041010

[B76] Wisnowski J. L. Blüml S. Paquette L. Zelinski E. Nelson M. D. Jr. Painter M. J. (2013). Altered glutamatergic metabolism associated with punctate white matter lesions in preterm infants. PLoS ONE 8:e56880 10.1371/journal.pone.0056880 23468888 PMC3582631

[B77] Woodin M. A. Ganguly K. Poo M.-M. (2003). Coincident pre- and postsynaptic activity modifies GABAergic synapses by postsynaptic changes in Cl transporter activity. Neuron 39, 807–820 10.1016/S0896-6273(03)00507-5 12948447

[B78] Yanai J. Bergman A. Shafer R. Yedwab G. Tabakoff B. (1981). Audiogenic seizures and neuronal deficits following early exposure to barbiturate. Dev. Neurosci. 4, 345–350 10.1159/000112774 7327097

[B79] Yanai J. Fares F. Gavish M. Greenfeld Z. Katz Y. Marcovici G. (1989). Neural and behavioral alterations after early exposure to phenobarbital. Neurotoxicology 10, 543–554 2696900

[B80] Yanai J. Rosselli-Austin L. Tabakoff B. (1979). Neuronal deficits in mice following prenatal exposure to phenobarbital. Exp. Neurol. 64, 237–244 10.1016/0014-4886(79)90265-6 428502

